# Genome-Wide Identification and Expression Profiling of Cytochrome P450 Monooxygenase Superfamily in Foxtail Millet

**DOI:** 10.3390/ijms241311053

**Published:** 2023-07-04

**Authors:** Xiaorui Li, Linlin Wang, Weidong Li, Xin Zhang, Yujia Zhang, Shuqi Dong, Xi’e Song, Juan Zhao, Mingxun Chen, Xiangyang Yuan

**Affiliations:** 1 College of Agronomy, Shanxi Agricultural University, Taigu 030801, China; lixiaorui@sxau.edu.cn (X.L.); wll18534272712@163.com (L.W.); li15110671274@163.com (W.L.); zx1150472698@163.com (X.Z.); zhangyujia202203@163.com (Y.Z.); dong-s-q@163.com (S.D.); sxndsxe@163.com (X.S.); 2 College of Agronomy, Northwest A&F University, Yangling 712100, China; cmx786@nwafu.edu.cn

**Keywords:** cytochrome P450, foxtail millet, expression analysis, abiotic stresses, herbicide

## Abstract

The cytochrome P450 monooxygenases (CYP450) are the largest enzyme family in plant metabolism and widely involved in the biosynthesis of primary and secondary metabolites. Foxtail millet (*Setaria italica* (L.) P. *Beauv*) can respond to abiotic stress through a highly complex polygene regulatory network, in which the SiCYP450 family is also involved. Although the CYP450 superfamily has been systematically studied in a few species, the research on the CYP450 superfamily in foxtail millet has not been completed. In this study, three hundred and thirty-one *SiCYP450* genes were identified in the foxtail millet genome by bioinformatics methods, which were divided into four groups, including forty-six subgroups. One hundred and sixteen genes were distributed in thirty-three tandem duplicated gene clusters. Chromosome mapping showed that SiCYP450 was distributed on seven chromosomes. In the SiCYP450 family of foxtail millet, 20 conserved motifs were identified. *Cis*-acting elements in the promoter region of *SiCYP450* genes showed that hormone response elements were found in all *SiCYP450* genes. Of the three hundred and thirty-one *SiCYP450* genes, nine genes were colinear with the *Arabidopsis thaliana* genes. Two hundred *SiCYP450* genes were colinear with the *Setaria viridis* genes, including two hundred and forty-five gene duplication events. The expression profiles of *SiCYP450* genes in different organs and developmental stages showed that *SiCYP450* was preferentially expressed in specific tissues, and many tissue-specific genes were identified, such as *SiCYP75B6*, *SiCYP96A7*, *SiCYP71A55*, *SiCYP71A61*, and *SiCYP71A62* in the root, *SiCYP78A1* and *SiCYP94D9* in leaves, and *SiCYP78A6* in the ear. The RT-PCR data showed that *SiCYP450* could respond to abiotic stresses, ABA, and herbicides in foxtail millet. Among them, the expression levels of *SiCYP709B4*, *SiCYP71A11*, *SiCYP71A14*, *SiCYP78A1*, *SiCYP94C3*, and *SiCYP94C4* were significantly increased under the treatment of mesotrione, florasulam, nicosulfuron, fluroxypyr, and sethoxydim, indicating that the same gene might respond to multiple herbicides. The results of this study will help reveal the biological functions of the SiCYP450 family in development regulation and stress response and provide a basis for molecular breeding of foxtail millet.

## 1. Introduction

The cytochrome P450 monooxygenases (CYP450s, CYPs) are heme proteins widely distributed in animals and plants [1], and their functions span from key structural components to the synthesis of signaling molecules and defense compounds [2]. The P450 enzyme system consists of cytochrome P450, NADPH-cytochrome P450, oxidoreductase (CPR), cytochrome b5, NADH-cytochrome b5, reductase (CBR), and phospholipids. Among them, the most important is cytochrome P450 protein, which is regarded as the terminal oxidase in the composition of the P450 enzyme system and is related to the differences and universality of substrates in the P450 enzyme system [3].

Since the first plant CYP450 was found in cotton in 1969 [4], more CYP450 sequences have been identified as more genomic sequences have become available. CYP450 genes have been identified in many plant species, including 273 in *Arabidopsis thaliana* [5], 355 in rice [2], 1285 in wheat, 263 in maize [6], 372 in sorghum, 316 in grape, 312 in *Populus trichocarpa* [7], etc. Notably, when the total number of CYP sequences contains pseudogenes and short fragments, the number is significantly increased, indicated the dynamic characteristics of CYPomes and the existence of multiple gene replications and losses [8].

In the primary amino acid sequence of CYP450s, only a few domains are highly conserved: the proline-rich membrane hinge, the sequence surrounding the cysteine which is the axial ligand to the heme, the I-helix (AGxD/ET) involved in oxygen binding, and the E-R-R triad consisting of the Glu and Arg of the K-helix consensus sequence (KETLR) and the Arg in the “PERF” consensus sequence [9]. Among these conserved domains, only the E-R-R triad and the cysteine in the heme-binding domain are conserved in the CYP450 sequences of all plants [10]. The classification and nomenclature of CYP450 family and subfamily genes are primarily based on peptide similarities. Two CYP450 proteins with sequence homology greater than 40% belong to the A-type family, and 55% belong to the non-A-type subfamily. In plants, A-type is included in the CYP71 clade, while non-A-type is divided into ten clades, including CYP51, CYP72, CYP74, CYP55, CYP86, CYP97, CYP710, CYP711, CYP727, and CYP746 [11,12,13].

CYP450s, as one of the largest enzyme protein families, play a vital role in plant development and physiological processes. For example, overexpression of *CYP78A9* in *Arabidopsis* led to large, seedless fruits [14], and loss of function of CYP78B5 in *japonica* rice led to larger embryos [15]. *Arabidopsis* CYP715 plays a key regulatory role in flower maturation, petal expansion, and volatile release [16]. CYP450s also respond to abiotic stresses such as drought, salinity, and low-temperature, and regulate plant hormone homeostasis. Reduced expression of *OsCYP707A7* in rice resulted in increased abscisic acid (ABA) content and antioxidant enzyme activity, thereby enhancing drought tolerance in rice [17]. Loss-of-function of *AtCYP709B3* in *Arabidopsis* resulted in delayed germination under NaCl treatment and enhanced salt tolerance [18]. Wheat *TaCYP81D5* enhanced salt tolerance by enhancing ROS signal transduction and scavenging [19]. The CYP97 family plays a key enzymatic role in the biosynthesis of the ABA precursor lutein [20], and CYP711As has been proven to be a strigolactone (SL) biosynthetic enzyme [21]. CYP701A26 had *ent*-kaurene oxidase activity with a mixed substrate that might be involved in the biosynthesis of maize gibberellin (GA) [22]. In addition, CYP450s plays an important role in the detoxification of xenobiotic chemicals and herbicides. Previous studies have shown that some *CYP450* genes encode CYP71, CYP72, CYP73, CYP81, and CYP749 proteins. They are responsible for acetyl-CoA carboxylase (ACCase) (sethoxydim), acetolactate synthase (ALS) (nicosulfuron), photosystem II (PSII) (florasulam), hydroxyphenyl pyruvate dioxygenase (HPPD) (mesotrione), and synthetic auxin herbicide (fluroxypyr) metabolic resistance [23,24,25,26,27,28,29,30,31,32]. *Arabidopsis* CYP76C1, CYP76C2, and CYP76C4 were capable of metabolizing herbicides monoterpene phenol and phenylurea [33], and CYP71A10 in tobacco was also capable of metabolizing phenylurea herbicides [25] Barley CYP81A63 metabolized three herbicides (tralkoxydim, diclofop-methyl, and pinoxaden) belonging to three different chemical classes of inhibitors of ACCase [29].

Foxtail millet is an ancient crop that originated in China, with a history of domestication and cultivation of more than 10,000 years. It has such outstanding advantages as resistance to dry climates, poor soil quality, and high efficiency of photosynthesis. At the same time, foxtail millet is rapidly developing into a model plant for C_4_ photosynthesis and functional gene discovery of Gramineae due to its outstanding characteristics such as a small genome, self-pollination, multiple single-spike seed setting, and easy use in the laboratory, therefore it will become a hot crop for functional gene research in plants in the future [34,35,36]. Although efforts have been made to study the functional characteristics of foxtail millet and SiCYP450s, little information is available about SiCYP450s in foxtail millet. Therefore, the *SiCYP450* gene family in foxtail millet has mainly been studied by using the methods of genomics and bioinformatics. The physicochemical properties, chromosomal distribution, gene structure, conserved motifs, and *cis*-acting elements of the SiCYP450 proteins were analyzed. The gene expression of the *SiCYP450* gene family was analyzed after various treatments, including three abiotic stresses (PEG, NaCl, and 4 °C), ABA, and five herbicides (sethoxydim, florasulam, nicosulfuron, mesotrione, and fluroxypyr). The results will help to further explore the function of SiCYP450s in foxtail millet and develop improved varieties.

## 2. Results

### 2.1. Identification and Nomenclature of SiCYP450 Genes

**Figure 1:**
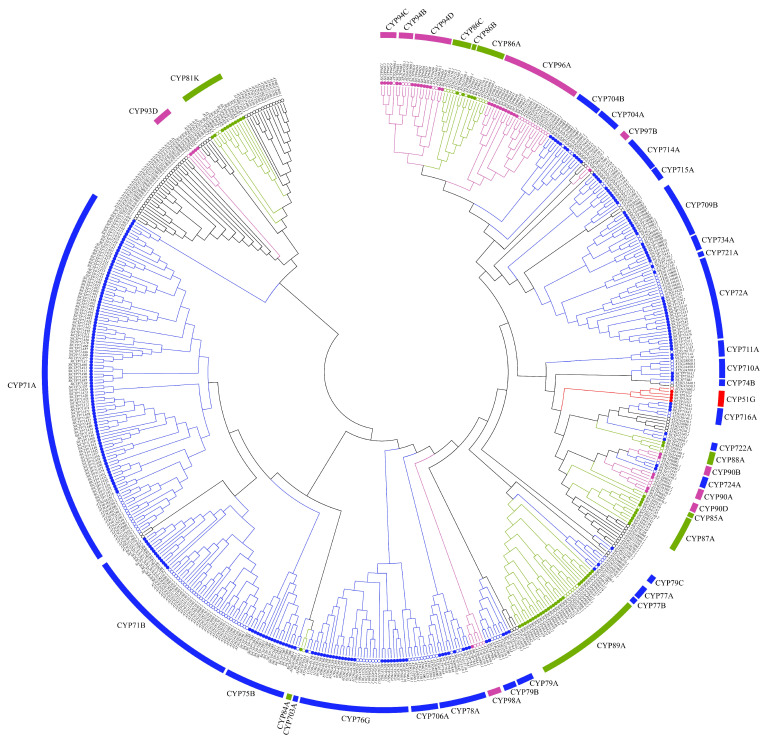
Phylogenetic tree of CYP450s from *Setaria italica* and *Arabidopsis thaliana*. The unrooted neighbor-joining (NJ) tree was constructed from *Setaria italica* (331) and *Arabidopsis thaliana* (234) using MEGA7.0 with 1000 bootstrap replicates. The names of groups are shown outside of the circle. Branch lines of subtrees are colored, indicating different CYP450 subgroups.

Three hundred and thirty-one full-length *SiCYP450* genes were identified in foxtail millet. The evolutionary relationship analysis was based on the 331 identified *CYP450* genes of *Setaria italica* and those of *Arabidopsis thaliana* (Figure 1 and Figure S1). The *SiCYP450* genes were classified and named according to the classification of *Arabidopsis*. All the *SiCYP450* genes were grouped into four groups (CYP5X, CYP7X, CYP8X, and CYP9X). Each group was further divided into several subgroups except CYP5X. CYP5X only has one subgroup (CYP51G), and there were only four genes (*SiCYP51G1*, *SiCYP51G2*, *SiCYP51G3*, and *SiCYP51G4*) in the CYP51G group. There were 26 subgroups in the CYP7X group, and the subgroup CYP71A had the most genes with 94 genes. The CYP8X and CYP9X groups had nine and ten subgroups, respectively. In total, there were 46 subgroups in four groups of *SiCYP450* genes. CYP71 was the largest family of A-type in foxtail millet (107 genes), while foxtail millet CYP72 was the largest family of non-A-type with 16 members (Table S1).

### 2.2. Physiochemical Properties of SiCYP450 Proteins

According to the physicochemical property assessments (Table S2), the lengths of the 331 SiCYP450 proteins varied markedly. The smallest protein was SiCYP71A84 (113 aa) with a molecular weight of 12.50 kDa, and the largest protein was SiCYP714A7 (950 aa) with a molecular weight of 106.58 kDa. Most proteins ranged in length from 400 aa to 600 aa. The isoelectric point ranged from 4.97 (SiCYP81K1) to 11.91 (SiCYP76G1) among the 331 SiCYP450 proteins. The isoelectric point of SiCYP75B2 was 7.00, which was the only neutral protein. The isoelectric point of 270 proteins was over 7.00, which indicated that over 81% of SiCYP450 proteins were basic proteins. The instability index ranged from 28.69 (SiCYP709B5) to 62.38 (SiCYP89A11), and a total of 75 genes were stable among the 331 genes, whereas all others were unstable. The aliphatic index ranged from 48.17 (SiCYP71A45) to 122.70 (SiCYP76G1). The grand averages of hydropathicity indicated that a total of 248 proteins were hydrophilic. Subcellular localization analysis predicted that the 331 SiCYP450 proteins were localized in the inner membrane, cytoplasmic, and periplasmic spaces. One hundred and seventy-one and one hundred and fifty-eight SiCYP450 proteins were localized in the cytoplasmic and inner membrane spaces, respectively. Only two proteins (SiCYP704A2 and SiCYP71A88) were localized in the periplasmic space.

### 2.3. Chromosome Mapping, Gene Tandem Duplication Analysis, and Syntenic Analysis

**Figure 2:**
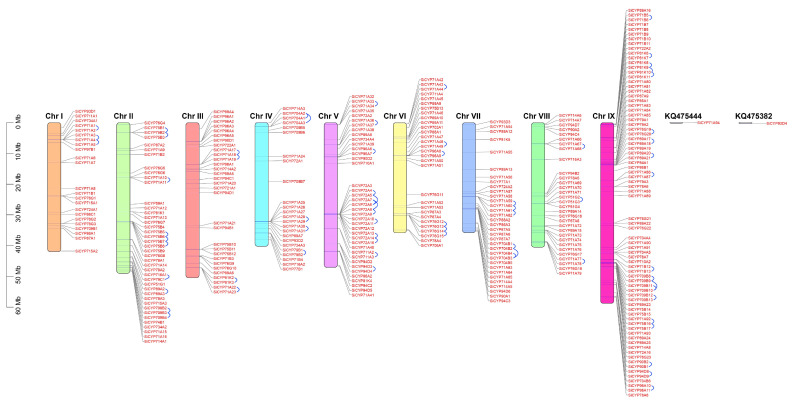
Chromosomal localization of *SiCYP450* genes. All putative *SiCYP450s* are shown on the chromosomes with red font. The chromosomal position of each *SiCYP450* was mapped according to the foxtail millet genome. Different colored bars represent different chromosomes. The chromosome numbers are shown at the top of each chromosome. The location of each *SiCYP450* gene is indicated by a grey line. Two *SiCYP450s* on the scaffold could not be anchored onto any specific chromosome. The scale is in megabases (Mb). The tandemly duplicated genes are joined with a blue curve.

To understand the distribution of *SiCYP450* genes, location information was used to locate the genes on the chromosomes. Among the three hundred and thirty-one *SiCYP450* genes, three hundred and twenty-nine were mapped onto nine chromosomes, while the other two genes (*SiCYP71A94* and *SiCYP93D4*) were not distributed on any of those nine chromosomes (Figure 2). Moreover, the distribution was not uniform, as nearly half of the *SiCYP450* genes were relatively distributed on chromosomes IX (77, 23.3%), II (41, 12.4%), and V (39, 11.8%). The minimum number, 23, was on chromosome I. Both chromosomes III and VIII had the same number, 31, of *SiCYP450* genes.

In this study, we identified 69 *SiCYP450* gene tandem duplication pairs, which corresponded to 116 genes (Figure 2 and Table S3). As shown in Figure 2 and Table S3, sixty-nine *SiCYP450* gene tandem duplication clusters were identified on nine chromosomes such as *SiCYP71A1* with *SiCYP71A2* and *SiCYP71A3* with *SiCYP71A4.* The maximum number of tandem duplications also occurred on chromosome Ⅸ with 19 gene pairs, followed by 13 genes pairs on chromosome V.

**Figure 3:**
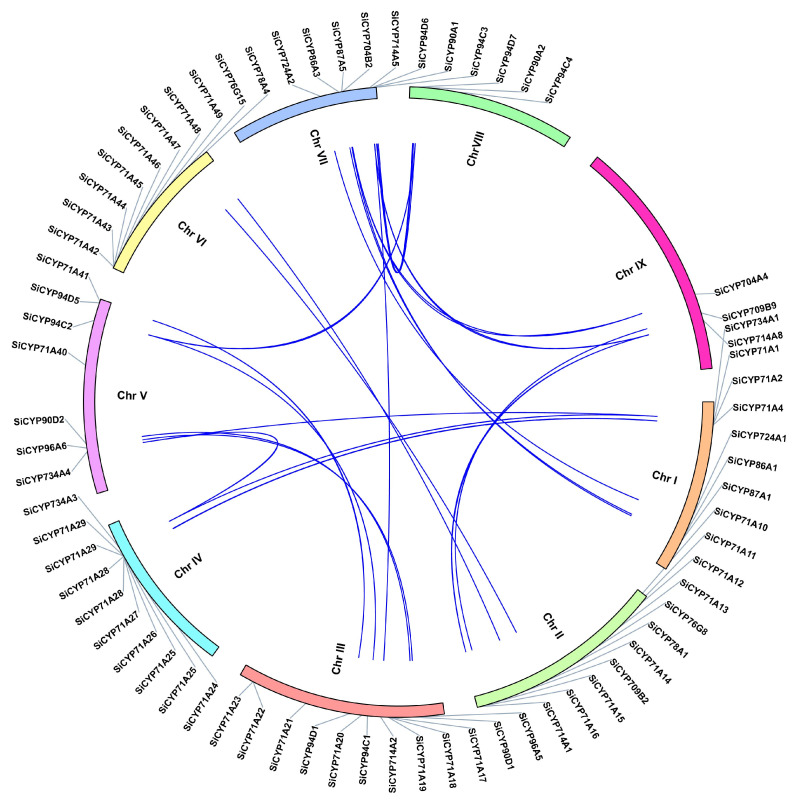
Synteny analysis of *SiCYP450* gene family in foxtail millet. Different colored bars represent different chromosomes. The location of each *SiCYP450* gene is indicated by a grey line. The blue line represents the synteny between two genes.

**Figure 4:**
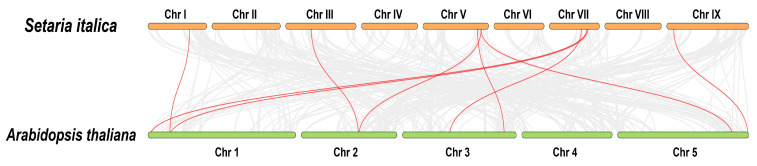
Synteny analyses of *CYP450* genes between *Setaria italica* and *Arabidopsis thaliana*. The orange and green bars represent the chromosomes of *Setaria italica* and *Arabidopsis thaliana*, respectively. The red line represents the synteny between two genes.

In addition, we analyzed the segmental duplications on different chromosomes. Forty-one genes were detected as twenty-four duplicated gene pairs (Figure 3). Therefore, these genes contribute substantially to the expansion of the *SiCYP450* gene family in *Setaria italica*. To understand the origin of the *SiCYP450* gene family, we also detected the duplication of *SiCYP450* genes with *Arabidopsis thaliana* and *Setaria viridis* genes (Figure 4 and Figure S2). Of the three hundred and thirty-one *SiCYP450* genes, nine genes were colinear with the *Arabidopsis* genes. Two hundred *SiCYP450* genes were colinear with the *Setaria viridis* genes, containing two hundred and forty-five gene duplication events. Some *SiCYP450* genes might have been produced by segmental and tandem duplications, and these replication events were the main driving force of *SiCYP450* gene evolution.

### 2.4. Gene Structure and Motif Composition Analysis

**Figure 5:**
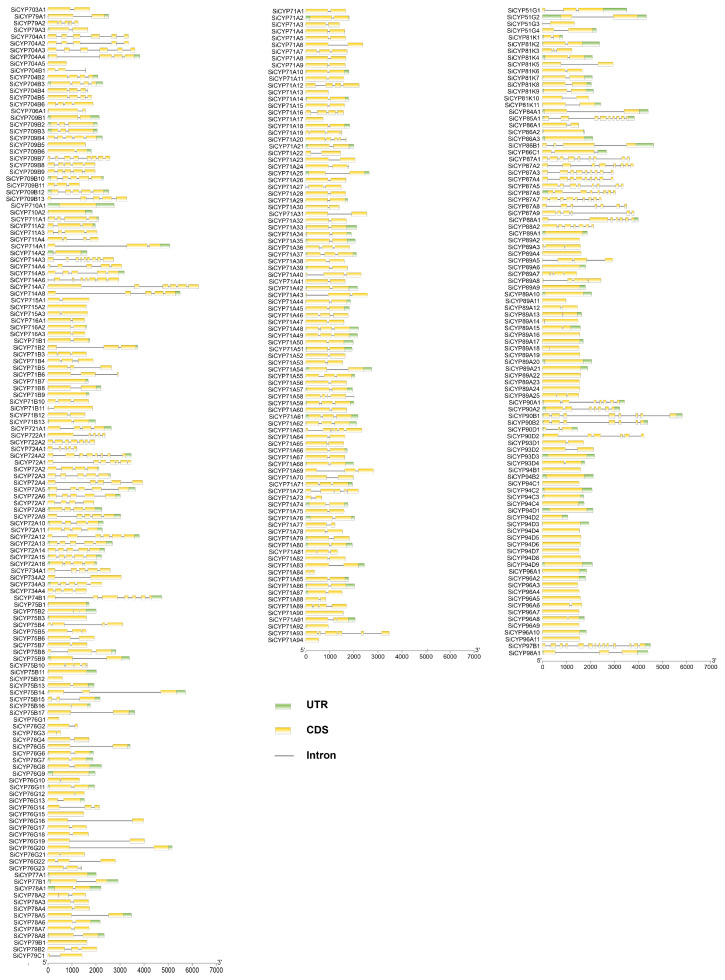
The gene structure of the *SiCYP450* gene family in foxtail millet. The green boxes and the yellow boxes represent the UTR and CDS. The gray lines represent the introns.

Based on the data of gene annotation, a gene structure map was produced (Figure 5 and Table S4). The numbers of introns and exons differed between genes. Sixty-three *SiCYP450* genes contained only one exon (i.e., *SiCYP79B1*, *SiCYP89A2*, and *SiCYP96A5*), and only seven genes comprised not less than nine exons (*SiCYP714A3*, *SiCYP85A1*, *SiCYP87A1*, *SiCYP87A3*, *SiCYP87A4*, *SiCYP87A7*, and *SiCYP97B1*). *SiCYP97B1* consisted of up to 14 exons. Overall, the gene structure of SiCYP450 was relatively simple. The gene structure in the four groups did not show obvious regularity.

**Figure 6:**
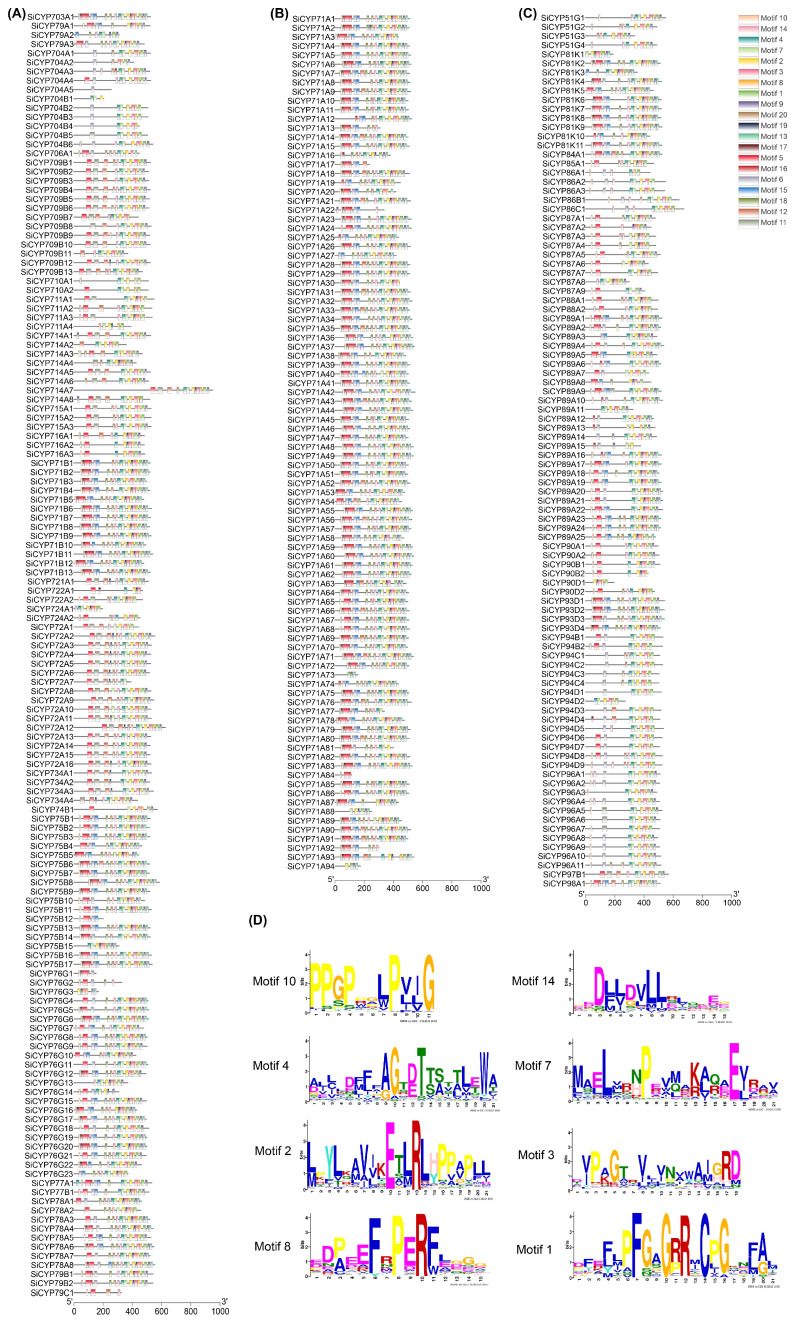
Schematic diagram of conserved motifs of *SiCYP450* gene family in foxtail millet. (**A**–**C**) Distribution of conserved motifs of SiCYP450s from different groups and subgroups. The conserved motifs are represented with boxes in the SiCYP450 proteins using MEME. Box size indicates the length of motifs. The gray lines represent the non-conserved sequences. The motifs, numbered 1–20, are displayed in different colored boxes. (**D**) Logo of the top eight motifs of *SiCYP450* gene family in foxtail millet.

To examine the characteristic regions of SiCYP450 proteins, twenty motifs were analyzed using the MEME database, and a conserved motif of SiCYP450 protein was produced (Figure 6). The results showed that motif 10 and motif 14 were highly conserved and existed in most genes. They might be characteristic conserved domains of the *SiCYP450* gene family. Motif 10 and motif 14 were composed of 11 and 15 amino acid sites, respectively, and contained multiple conserved sites. There were only fifty *SiCYP450* genes containing motif 11, including forty-five genes in the CYP7X group, one gene in the SiCYP89A subgroup, three genes in the SiCYP96A subgroup, and one gene in the SiCYP97B subgroup. The motifs of different subfamilies were different. There were more members in the CYP71A subgroup, and motif 16 was found in 88 CYP71A protein sequences, but there was no motif 16 in the CYP709B, CYP710A, CYP711A, CYP714A, CYP715A, CYP716A, CYP72A, CYP721A, CYP722A, CYP724A, CYP734A, CYP74B, CYP77B, CYP78A, and CYP79C subgroups of the CYP7X group. Motif 2, motif 3, and motif 8 were found in all CYP9X protein sequences. In CYP5X and CYP8X classes, there were multiple identical motifs.

### 2.5. Promoter Element Analysis of SiCYP450 Genes

**Figure 7:**
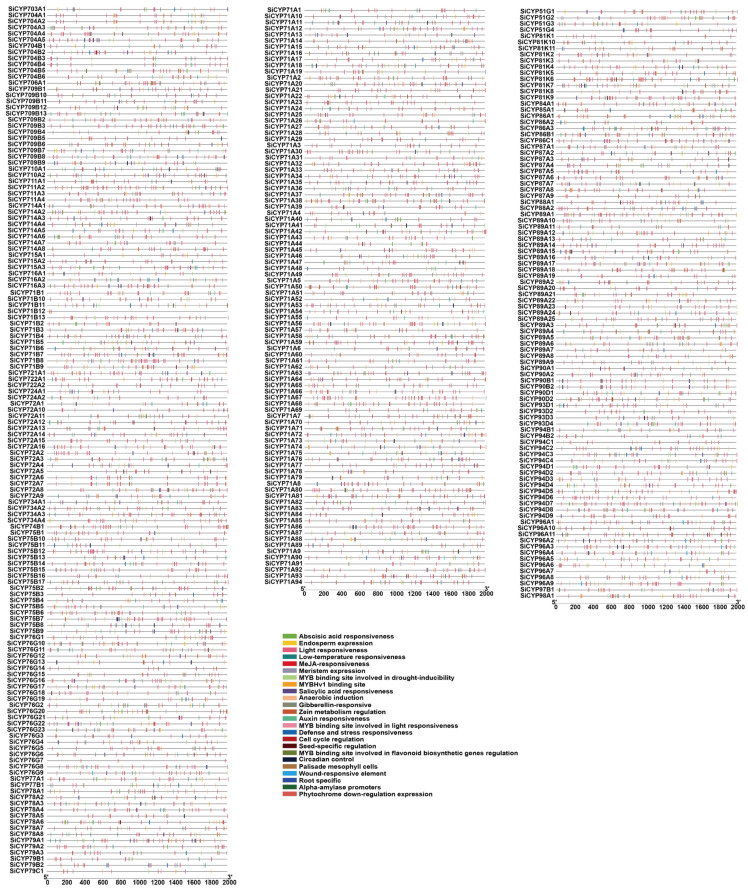
Distribution of *cis*-acting elements of *SiCYP450* gene family in foxtail millet.

TBtools was used to extract a 2000 bp upstream promoter of the *SiCYP450* gene family as a promoter sequence, and the PlantCARE database was used for *cis*-acting element analysis (Figure 7 and Table S5). The results showed that the *SiCYP450* gene family contains many types of promoter elements. The majority of *SiCYP450* gene promoters were found to most light response elements. At the same time, many hormone-related functional elements were analyzed, including abscisic acid responsiveness, MeJA responsiveness, gibberellin responsiveness, auxin responsiveness, and salicylic acid responsiveness. Among them, there were more *cis*-acting elements related to abscisic acid and MeJA responsiveness and fewer *cis*-acting elements related to auxin, salicylic acid, and gibberellin responsiveness. Additionally, many stress-related response elements were analyzed, including anaerobic induction, low-temperature response, defense and stress, and wound response. The wound-responsive element was only analyzed in several genes, and the number was small. *Cis*-acting elements related to growth and development were also analyzed, including endosperm expression, meristem expression, zein metabolism regulation, palisade mesophyll cells, seed-specific regulation, circadian control, root-specific elements, and cell cycle regulation. The light response elements were found in the promoter regions of 330 *SiCYP450* genes, and eighty *SiCYP450* genes in the defense and stress response elements. All three hundred and thirty-one *SiCYP450* gene promoters contained hormone response *cis*-acting elements. In addition, sixty-five *cis*-acting elements were identified in the promoter of the *SiCYP71A80* transcript. However, only three *cis*-elements were found in the promoter of *SiCYP76G7*, and only four *cis*-elements were found in *SiCYP71B12*. In conclusion, the *SiCYP450* genes were involved in light response, hormone response, stress response, and plant growth and development.

### 2.6. Expression Profiles of SiCYP450 Genes in Various Organs and Developmental Stages

**Figure 8:**
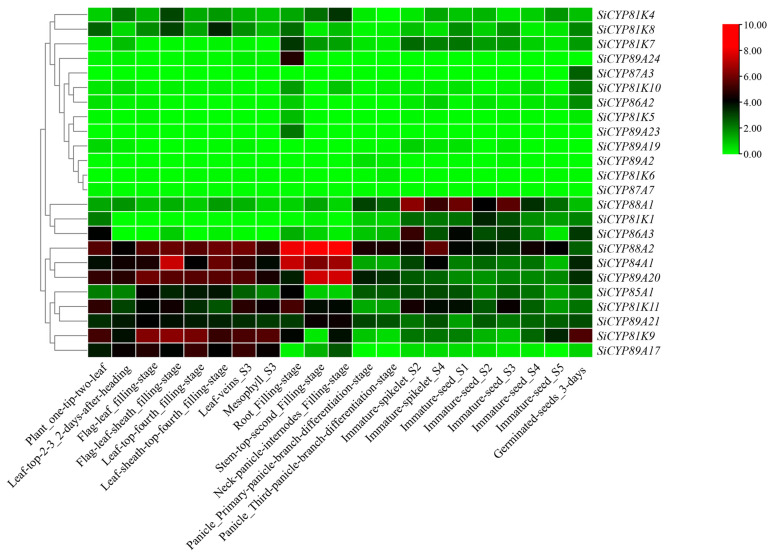
Relative expression patterns (FPKM value) of *CYP8X* group genes involved in various tissues and different developmental stages of foxtail millet. The color bar represents log_2_ expression levels (FPKM), and the red and green colors indicate the high and low gene expression levels, respectively.

In order to understand the expression patterns of *SiCYP450* genes in various organs and development stages, we studied the expression profiles of *SiCYP450s* in roots, stems, leaves, ears, and seeds at different development stages (Figure 8, Figures S3–S5 and Table S6). By comparing the gene expression profiles of different organs, the *SiCYP450* transcripts of foxtail millet were detected in all tissues, but the transcription levels in root and leaf were the highest. Among them, the transcription levels of *SiCYP75B6*, *SiCYP88A2*, and *SiCYP71A48* reached the peak in the roots, indicating that they exhibited the preferential expression pattern in roots. *SiCYP97B1* and *SiCYP98A1* were both expressed specifically and in large amounts in different tissues during the whole development process. However, the expressions of *SiCYP76G4*, *SiCYP87A7*, *SiCYP71B5*, *SiCYP71A66*, and *SiCYP71A82* were extremely low or they were not expressed. The results also showed that the expression of *SiCYP450* genes in different tissues was different, indicating that some *SiCYP450s* were tissue-specific genes. For example, *SiCYP450s* were expressed more in the leaves than in other organs regarding *SiCYP78A1* and *SiCYP94D9*. In addition, the expression patterns of the same gene at different stages of development were also different. The expression levels of *SiCYP88A2*, *SiCYP84A1*, and *SiCYP89A20* in all tissues during the filling stage were high, in particular the expression of *SiCYP88A2* was extremely high in the root, neck panicle internodes, and stem-top-second during the filling stage.

### 2.7. Relative Expression Patterns of 14 SiCYP450s under Abiotic Stress, ABA, and Herbicide Treatments

**Figure 9:**
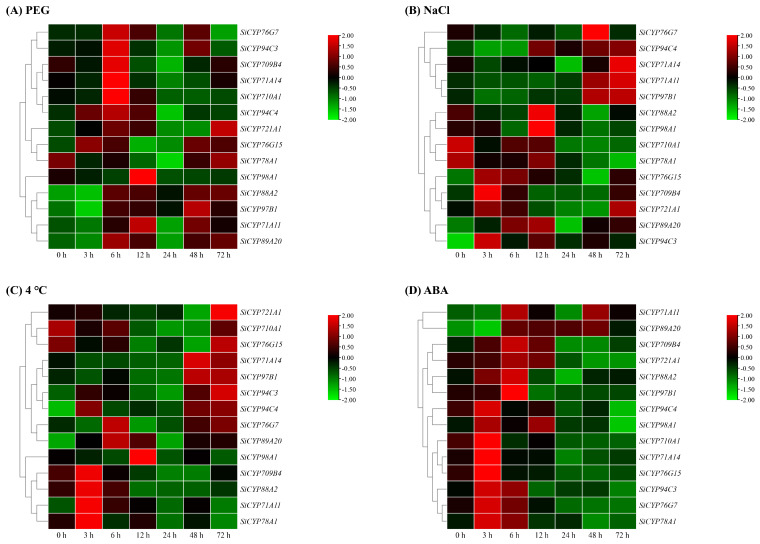
Relative expression levels of 14 *SiCYP450* genes under abiotic stress and ABA treatment were analyzed by RT-PCR. Relative expression patterns of 14 *SiCYP450* genes were analyzed under osmotic (20% PEG 6000) (**A**), salt (200 mM NaCl) (**B**), and low-temperature stress (4 °C) (**C**) and 100 μM ABA (**D**). The heat map of *SiCYP450* expression was constructed by TBtools, expression value was standardized by log_2_. The expression level of the target gene at 0 h was used as a control.

**Figure 10:**
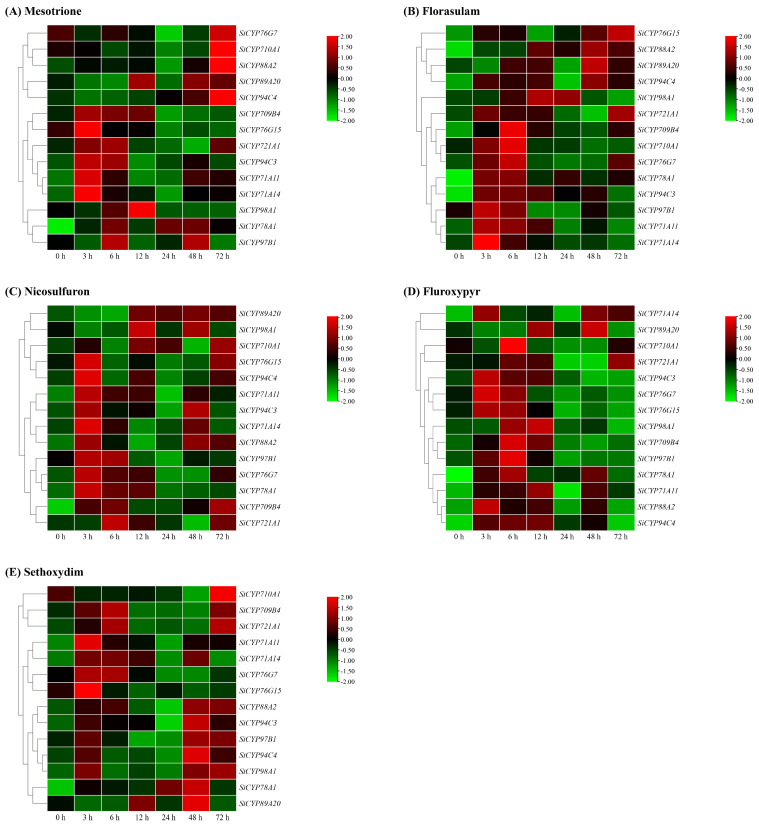
Relative expression levels of 14 *SiCYP450* genes under the treatment of five herbicides were analyzed by RT-PCR. Relative expression patterns of 14 *SiCYP450* genes were analyzed under 150 g ha^−1^ mesotrione (**A**), 4.5 g ha^−1^ florasulam (**B**), 60 g ha^−1^ nicosulfuron (**C**), 210 g ha^−1^ fluroxypyr (**D**), and 187.5 g ha^−1^ sethoxydim (**E**). The heat map of the *SiCYP450* expression was constructed by TBtools, and the expression value was standardized by log_2_. The expression level of the target gene at 0 h was used as a control.

Drought, salt, low-temperature, ABA, and herbicides are extremely threatening stresses for most plants, but little is known about the response of *SiCYP450* genes in foxtail millet to these stresses. Therefore, we selected 14 *SiCYP450* genes and analyzed their expression patterns in foxtail millet under osmotic (20% PEG 6000), salt (200 mM NaCl), low-temperature stress (4 °C), 100 μM ABA, and herbicide (mesotrione, florasulam, nicosulfuron, fluroxypyr, and sethoxydim) treatments using RT-PCR (Figure 9 and Figure 10). Under osmotic stress treatment, the expression levels of all genes except *SiCYP78A1* were increased to different degrees (Figure 9A). Among them, the up-regulation of *SiCYP710A1* expression was the most significant, and its expression reached the peak at 6 h, which was 14.7 times as high as that at 0 h. Similarly, the expression levels of *SiCYP709B4*, *SiCYP71A14*, *SiCYP76G7*, *SiCYP76G15*, *SiCYP89A20*, *SiCYP94C3*, and *SiCYP94C4* peaked at 6 h. The expression levels of *SiCYP71A11* and *SiCYP98A1* peaked at 12 h, *SiCYP721A1* and *SiCYP88A2* peaked at 72 h, and only *SiCYP97B1* peaked at 48 h. The expression levels of *SiCYP88A2* and *SiCYP89A20* remained high after 6 h of osmotic stress treatment.

Under salt stress treatment, the expression levels of *SiCYP709B4*, *SiCYP71A11*, *SiCYP721A1*, *SiCYP76G15*, *SiCYP89A20*, *SiCYP94C3*, and *SiCYP94C4* were significantly higher than those in the control group (Figure 9B). The expression levels of *SiCYP710A1* and *SiCYP78A1* were both inhibited and were lower than that in the control group. The expression peak for *SiCYP709B4*, *SiCYP76G15*, and *SiCYP94C3* appeared at 3 h and up-regulated 18.1-fold, 8.9-fold, and 5.1-fold compared to the control group. At 48 h, the expression levels of *SiCYP76G7* were significantly higher than those in the control group. At 72 h, expression levels of *SiCYP71A11*, *SiCYP71A14*, *SiCYP721A1*, *SiCYP94C4*, and *SiCYP97B1* were significantly higher than those in the control group.

Under low-temperature treatment, the expression of *SiCYP710A1* was down-regulated, and the expressions of *SiCYP71A11*, *SiCYP76G7*, *SiCYP89A20*, *SiCYP94C3*, and *SiCYP94C4* were up-regulated. The expression levels of *SiCYP709B4* and *SiCYP88A2* increased first and then decreased. The *SiCYP709B4*, *SiCYP71A11*, *SiCYP78A1*, *SiCYP88A2*, and *SiCYP94C4* genes showed an expression peak at 3 h. The expression peak of *SiCYP721A1* and *SiCYP94C3* appeared at 96 h, which was about 13.5 times that of 0 h (Figure 9C).

These *SiCYP450* genes also were responsive to ABA (Figure 9D). During the early stage of ABA treatment, the expression levels of *SiCYP709B4*, *SiCYP710A1*, *SiCYP71A14*, *SiCYP76G7*, *SiCYP76G15*, *SiCYP78A1*, *SiCYP88A2*, *SiCYP94C3*, and *SiCYP94C4* increased. However, the expression level of *SiCYP89A20* after 3 h of ABA treatment was higher than that in the control group.

Additionally, they might be responsive to at least one herbicide. Under the treatment of mesotrione, the expression levels of *SiCYP71A11*, *SiCYP71A14*, *SiCYP78A1*, *SiCYP94C3*, and *SiCYP94C4* were significantly increased, and the peak expression levels were 14.5-fold, 15.0-fold, 7.5-fold, 8-fold, and 13.3-fold higher than those in the control group, respectively. Among them, the expression of *SiCYP78A1* was high during the whole treatment period, while *SiCYP71A11* and *SiCYP71A14* increased sharply in the early treatment period and then decreased rapidly. The expression levels of *SiCYP710A1* and *SiCYP76G7* were slightly increased after 72 h treatment with mesotrione, but the increase was insignificant (Figure 10A).

Under florasulam treatment, *SiCYP709B4*, *SiCYP78A1*, *SiCYP88A2*, and *SiCYP94C3* were induced more significantly during the treatment period, exhibiting a higher expression difference compared with 0 h. The expression peak of *SiCYP71A11*, *SiCYP71A14*, and *SiCYP78A1* at 3 h was about 14.2 times that of 0 h. The expression of *SiCYP709B4*, *SiCYP710A1*, *SiCYP76G7*, and *SiCYP94C3* peaked at 6 h, and the expression levels of *SiCYP709B4* and *SiCYP76G7* at 6 h were about 15.7-fold and 14.7-fold higher than those at 0 h. The expression peak of *SiCYP721A1* and *SiCYP76G15* appeared at 72 h, which was about 16.1 times that of 0 h (Figure 10B).

Under nicosulfuron treatment, the expression of *SiCYP709B4* remained at a high level and reached a peak of about 10.7 times that of 0 h after 72 h treatment. The expression levels of *SiCYP76G15* and *SiCYP78A1* were higher in the early treatment stage and reached peak values 7.5 times and 9.7 times higher than 0 h after 3 h treatment, respectively. However, the expression of *SiCYP89A20* began to increase after 6 h of nicosulfuron treatment (Figure 10C).

After fluroxypyr treatment for 3 h, the expression levels of *SiCYP76G7*, *SiCYP76G15*, *SiCYP88A2*, and *SiCYP94C3* reached the peak, which were higher than those in the control group. At 6 h, *SiCYP709B4*, *SiCYP710A1*, *SiCYP78A1*, and *SiCYP97B1* reached the peak, and the expression of *SiCYP709B4* was about 16.2 times that of 0 h. At 12 h, *SiCYP71A11*, *SiCYP94C4*, and *SiCYP98A1* were higher than in the control group (Figure 10D).

The expression levels of 14 *SiCYP450* genes were increased to different degrees under the sethoxydim treatment (Figure 10E). The expression levels of *SiCYP709B4*, *SiCYP721A1*, *SiCYP94C4*, *SiCYP97B1*, and *SiCYP98A1* were temporarily up-regulated, then decreased, and then increased after sethoxydim treatment. However, the expression of *SiCYP78A1* remained at a high level and peaked at about 13.2 times that of 0 h after 48 h of treatment. In summary, the RT-PCR assay showed that there existed many different *SiCYP450* gene expression patterns in foxtail millet under various stresses, and the diversity of expression patterns was related to the diversity of gene functions.

## 3. Discussion

### 3.1. Identification and Analysis of SiCYP450 Genes in Foxtail Millet

In recent years, researchers have continuously isolated new CYP450 families and genes from plants, and the constructed CYP450 database covers animals, plants, fungi, and other organisms, including 66 plants such as *Arabidopsis*, tomato, corn, and barley [37]. The *Arabidopsis* genome contains 286 *CYP450* genes (including unclassified genes), which are divided into 47 families, while rice, wheat, and maize have 45, 45, and 43 CYP450 families, respectively [2,5,6,37]. In this study, we identified 331 possible *SiCYP450* genes in foxtail millet (Figure 1). Systemic evolution analysis showed that 331 possible *SiCYP450* genes were assigned to 35 gene families. The numbers of members of the families SiCYP703, SiCYP721, SiCYP84, SiCYP85, and SiCYP88 in foxtail millet were the same as those of *Arabidopsis thaliana*, but foxtail millet did not contain the families CYP701, CYP702, CYP705, CYP707, CYP708, CYP712, CYP718, CYP720, CYP73, CYP735, CYP82, and CYP83 existing in *Arabidopsis*. In addition, studies have shown that only two-thirds of the *CYP450* gene families are common in *Arabidopsis* and rice, which might be related to the differences in *CYP450* genes among species. CYP71, the largest A-type family in foxtail millet, has 107 genes (Table S1), which was the same as the research results of other plants such as *Arabidopsis* (54 genes) [5], wheat (404 genes), and corn (56 genes) [6]. In addition, the syntenic analysis showed that 200 of 331 *SiCYP450* genes were colinear with *Setaria viridis* genes, including 245 gene duplication events, indicating that *Setaria italica* and *Setaria viridis* genes have a close genetic relationship.

The exon–intron distribution was analyzed to further detect structural features of *SiCYP450* genes. Figure 5 shows that the number of introns in *SiCYP450* family genes varied from 0 to 13, while 0 to 14 in rice [2], 0 to 15 in wheat [6], and 0 to 14 in maize [6]. This phenomenon suggested that *PCY450s* in foxtail millet show high gene structure diversity. A total of one hundred and thirty-seven (41.38%) *SiCYP450* genes with one intron accounted for the largest proportion, followed by sixty-three (19.03%) and forty-seven (14.2%) genes, possessing zero and two introns. The conserved motifs of SiCYP450 proteins in foxtail millet were analyzed to explore the similarity and diversity of motif compositions. A total of 20 distinct motifs, named motifs 1–20, were detected using the MEME online program (Figure 6). As displayed schematically in Figure 6, SiCYP450s within the same group or subgroup shared similar motif compositions, except several incomplete proteins, such as SiCYP704A5, SiCYP704B1, SiCYP76G1, SiCYP76G2, SiCYP79C1, and so on. Motif 11 was mainly scattered in CYP7X groups. In plants, motifs specific to populations might participate in specific biological processes, and each family or subfamily of the *CYP450* gene might be responsible for specific biological processes [38].

The *cis*-acting elements in the promoter are essential to gene expression, and are an essential component of its function [39]. In the study, various *cis*-acting elements were found in 331 of 331 *SiCYP450* genes (Table S5 and Figure 7). Many *cis*-acting elements were related to the response of hormones and biotic stresses, including MeJA, ABA, SA, GA, auxin, zein, and fungus. All *cis*-acting elements of the *SiCYP450* gene promoter included hormone-responsive elements. Additionally, some elements involved in various abiotic stresses, such as light, wounds, cold, heat, anaerobic induction, and drought were identified in a large number of *SiCYP450* genes. A total of 330 light-responsive elements were distributed in almost all of the *SiCYP450s*. Some elements were also observed in genes that might regulate the expression of different tissues in foxtail millet development. MBSI, the MYB binding site involved in the regulation of flavonoid biosynthesis genes, was present in ten *SiCYP450* genes, suggesting that these ten genes might regulate flavonoid metabolism. Another specific MYB binding site involved in drought response was identified in 216 genes, indicating that most *SiCYP450s* appeared to be involved in drought stress response. Notably, all members analyzed contained three or more cis-elements. Our analysis and previous studies have shown that the *SiCYP450* genes were involved in transcriptional regulation of plant growth and stress responses [8,13,40].

### 3.2. Expression Analysis of CYP450s during Development

Cytochrome CYP450 protein is widely believed to be involved in many developmental events through the biosynthesis and catabolism of plant hormones and other secondary compounds. For example, the biosynthesis and catabolism of brassinolides (BRs) required the involvement of CYP450s, including CYP500A, CYP90B, CYP90C, CYP90D, CYP724B, etc. [41,42]. AtCYP703A2, AtCYP86C3, OsCYP704B2, and OsCYP703A3 catalyzed the hydroxylation of mid-chain and long-chain fatty acids during xenobiotic pollen synthesis [43,44]. *OsCYP87A6* is a major auxin-responsive gene that in turn affects the local auxin levels, which are crucial for pollen development [45,46]. In our study, a portion of CYP450 was extensively expressed in foxtail millet tissues, such as *SiCYP97B1* and *SiCYP98A1* (Figure 8). The expression levels of *SiCYP75B6*, *SiCYP96A7*, *SiCYP71A55*, *SiCYP71A61*, and *SiCYP71A62* were higher in roots. The expression levels of *SiCYP78A1* and *SiCYP94D9* in leaves and *SiCYP78A6* in panicles were higher. Studies have shown that the expression of many *CYP450* genes in *Arabidopsis thaliana* is also tissue-specific. For example, *CYP88A3* and *CYP706A2* had high expression levels in leaf tissues, *CYP77A6* was specifically expressed only in flowers, *CYP708A2* was specifically expressed only in roots, and *CYP81F4* and *CYP83B1* were specifically expressed in vegetative tissues [5,47]. It seemed that each gene plays an important role in different tissues or species. This motivated us to assess their potential importance in controlling organ specificity.

*CYP450* genes have been reported to be involved in stress response. Wheat *TaCYP81D2*, *TaCYP81D3*, *TaCYP81D1*, and *TaCYP81D5* expressions were up-regulated under salt stress [19]. During dehydration or rehydration, the expression of *CYP707A1*, *CYP707A3*, and *CYP707A4* in *Arabidopsis* was higher, while *CYP707A2* was less expressed [48]. Expression of *Arabidopsis AtCYP709B3* was up-regulated after 24 h of salt stress treatment [18]. In this study, among the 14 adversity-related foxtail millet *SiCYP450* genes, 13, 12, 13, and 14 *SiCYP450* genes could be induced and expressed by osmotic stress, salt stress, low-temperature stress, and ABA, respectively, indicating that they have important biological functions in responding to different stresses. More importantly, the expressions of *SiCYP709B4*, *SiCYP71A11*, *SiCYP71A14*, *SiCYP721A1*, *SiCYP76G7*, *SiCYP76G15*, *SiCYP88A2*, *SiCYP89A20*, *SiCYP94C3*, *SiCYP94C4*, *SiCYP97B1*, and *SiCYP98A1* could be induced by osmotic stress, salt stress, low-temperature stress, and ABA and they showed overlapping expression characteristics, which indicated their functional pleiotropic effects in different abiotic stresses and ABA. In order to further demonstrate transcription regulation, cis-acting sequence analysis showed that the genes of *SiCYP721A1*, *SiCYP88A2*, *SiCYP89A20*, *SiCYP94C3*, *SiCYP97B1,* and *SiCYP98A1* all contained abiotic stress response elements such as drought, low-temperature, and abscisic acid response elements (Figure 7 and Table S5).

CYP450s have been the center of herbicide metabolism research due to their ability to confer crop selectivity and weed resistance [49]. The discovery of genes that metabolize herbicide resistance in plants was a challenging task because plants possess hundreds of CYP450s with varying substrate specificities [50]. Over the past 20 years, some CYP450s from different plant species have been found to have herbicide metabolic functions, some of which have been shown to play key roles in plant herbicide susceptibility. For example, wheat CYP71C6v1 [51] and rice CYP81A6 [27] were capable of metabolizing some ALS- and PSII-inhibiting herbicides. Transgenic rice overexpressing the *Lolium rigidum CYP81A10v7* gene could metabolize ACCase and ALS-inhibiting herbicides [52]. In addition, tobacco CYP81B2 and CYP71A11 [53] and soybean CYP71A10 [25] were also capable of metabolizing urea herbicides. The CYP76 family of *Arabidopsis* was capable of metabolizing terpene alcohol compounds and phenylurea herbicides [33]. This study found that the expression levels of all 14 *SiCYP450* genes were up-regulated under florasulam treatment, indicating that these genes might be involved in the metabolism of florasulam. The expressions of *SiCYP709B4*, *SiCYP71A11*, *SiCYP71A14*, *SiCYP78A1*, *SiCYP94C3*, and *SiCYP94C4* were significantly increased under the treatments of mesotrione, florasulam, nicosulfuron, fluroxypyr, and sethoxydim, indicating that the same gene might respond to multiple herbicides. This was similar to the fact that maize P450 (*Nsf1*) has been reported to be capable of metabolizing a variety of herbicides, including ALS-, HPPD-, protoporphyrinogen oxidase-, and PSII-inhibiting and synthetic auxin herbicides [54]. This study indirectly proved the role of *SiCYP450* genes in herbicide metabolic resistance, and has potential application value for further research on *CYP450* genes in the production of a variety of herbicide-resistant crops.

## 4. Materials and Methods

### 4.1. SiCYP450 Gene Identification, Phylogenetic Analysis, and Physicochemical Properties

HMMER 3.0 software (Boston, MA, USA) was used to identify *SiCYP450* genes, and the P450 domain sequence (PF00067) was downloaded from the Pfam database (https://pfam.xfam.org/, accessed on 7 June 2022). The *Setaria italica* genome data were downloaded from the NCBI database (https://www.ncbi.nlm.nih.gov/genome/?term=Setaria+italica+, accessed on 14 August 2021). The National Center for Biotechnology Information conserved domain database (https://www.ncbi.nlm.nih.gov/Structure/cdd/wrpsb.cgi, accessed on 7 June 2022) was used to detect P450 domains which were then mapped to the conserved domain. A phylogenetic tree was constructed using MEGA 7 software (Mega Limited, Auckland New Zealand) by the neighbor-joining method (bootstraps = 1000). *Arabidopsis* P450 protein sequences were downloaded from the TAIR database (https://www.arabidopsis.org/, accessed on 27 October 2020). Physicochemical properties and subcellular localizations were predicted using ExPASy-ProParam (https://web.expasy.org/protparam/, accessed on 9 June 2022) and the CELLO (http://cello.life.nctu.edu.tw/, accessed on 9 June 2022), respectively.

### 4.2. Gene Structure, Motif Compositions, Gene Synteny, and Promoter Analysis of SiCYP450 Genes

The gene structure map was produced, and an intron–exon map was compiled based on the *Setaria italica* genome annotation information using TBtools [55]. The protein motif analysis was carried out with the MEME database (http://meme-suite.org/tools/meme, accessed on 9 June 2022). Chromosomal positions of *SiCYP450* genes were analyzed by TBtools, and MCScanX was used to detect colinear regions between *SiCYP450* genes, as well as colinear blocks of *SiCYP450* genes with *Setaria viridis* and *Arabidopsis thaliana* genes. The *Setaria viridis* genome data were downloaded from the NCBI database (https://www.ncbi.nlm.nih.gov/genome/?term=Setaria+viridis, accessed on 14 August 2021). The *Arabidopsis thaliana* genome data were downloaded from the TAIR database (https://www.arabidopsis.org/, accessed on 27 October 2020). The upstream 2.0 kb DNA sequences of *SiCYP450* genes were submitted to the PlantCARE database (http://bioinformatics.psb.ugent.be/webtools/plantcare/html/, accessed on 17 June 2022) to predict cis-regulatory elements. All the above were visualized using TBtools [55].

### 4.3. Expression Profiles of SiCYP450 Genes

The FPKM values of *SiCYP450* genes in different tissues such as roots, stems, and leaves were extracted from the Multi-omics Database for *Setaria italica* (http://foxtail-millet.biocloud.net/home, accessed on 19 April 2022), and the expression heat maps of *SiCYP450* genes were drawn by TBtools software (Guangzhou, Guangdong, China).

### 4.4. Plant Material

Foxtail millet variety Jingu 21 was utilized in the experiment. The seeds were sterilized in 75% ethanol (*v*/*v*) for five minutes, and rinsed with distilled water. Then, the seeds were germinated in a box with a temperature of 26 °C, relative humidity of 65%, and a day/night cycle of 16 h/8 h. Two days later, the germinated seedlings were transferred into black plastic pots filled with half-strength Hoagland solution for further cultivation. For osmotic stress, salt stress, and ABA treatments, 12-day-old seedlings were treated with 1/2 Hoagland solution containing 20% PEG 6000, 200 mM NaCl, and 100 µM ABA, respectively. For low-temperature stress treatment, the 12-day-old seedlings were placed in a low-temperature (4 °C) incubator to develop. For herbicide treatments, the 12-day-old seedlings were sprayed with 150 g ha^−1^ mesotrione, 4.5 g ha^−1^ florasulam, 60 g ha^−1^ nicosulfuron, 210 g ha^−1^ fluroxypyr, and 187.5 g ha^−1^ sethoxydim by a spray tower. The seedlings in 1/2 Hoagland solution without treatment at 26 °C were regarded as controls. The leaves of the seedlings were sampled at 0 h, 3 h, 6 h, 12 h, 24 h, 48 h, and 72 h after 20% PEG 6000, 200 mM NaCl, 100 µM ABA, low-temperature (4 °C), and the five herbicide treatments. All samples were immediately frozen in liquid nitrogen and stored in a −80 °C refrigerator.

### 4.5. RNA Isolation and RT-PCR

The RNA was isolated from leaves using a TRIzol kit (Accurate Biotechnology, Changsha, China). The quality of RNA samples was verified by agarose gel electrophoresis (Figure S6). Then, the cDNA was synthesized using a reverse transcription kit (Accurate Biotechnology, Changsha, China), and real-time PCR was carried out using the SYBR Green dye method (Accurate Biotechnology, Changsha, China). The PCR primers were designed using Primer Premier 5 software (Vancouver, BC, Canada), and the primers are listed in Table S7. The RT-PCR reaction was accomplished in a Bio-Rad CFX system, with a 20 µL reaction system containing 10 µL of 2× SYBR Green Pro Taq HS premix, 2 µL of cDNA, 0.4 µL of each of forward primer and reverse primer, and 7.2 µL of RNase free water. The reaction conditions of RT-PCR were according to the instructions. The melting curve analysis of primers used for RT-PCR is presented in Figure S7. *SiActin* (SETIT_026509mg) was used as an internal standard, and the expression levels of each gene were calculated by the 2^−∆∆Ct^ method [56]. The heat map of *SiCYP450* expression was constructed by TBtools, the expression value was standardized by log_2_. Three biological replicates were conducted for the transcript profiles of genes. Each independent experiment was repeated at least three times.

### 4.6. Statistical Analysis

The data were first analyzed using Microsoft Office Excel 2016. The expression value was standardized by log_2_ in RT-PCR analysis. The figures were generated using the GraphPad Prism 7 (Boston, MA, USA), TBtools (Guangzhou, Guangdong, China), and Adobe Illustrator 2019 software (San Jose, CA, USA).

## 5. Conclusions

In this study, the SiCYP450 family of foxtail millet was first summarized, including phylogeny, physical and chemical properties of the proteins, gene structure, chromosomal localization, duplicated events, *cis*-acting elements, and gene expression pattern, which provide essential characteristics for the *SiCYP450* genes of foxtail millet. We identified three hundred and thirty-one *SiCYP450* genes in the foxtail millet genome, which were classified into four groups, including forty-six subgroups. Analysis of gene duplication events indicated that a large number of segmental duplication events and tandem duplications occurred during the expansion of the SiCYP450 family. The expression profile analyses of *SiCYP450* genes showed that most of the *SiCYP450* genes were highly expressed in roots and leaves. The RT-PCR analysis of 14 *SiCYP450* genes from different subfamilies confirmed that the *SiCYP450* genes extensively participated in abiotic stress, ABA, and herbicide responses, including response to drought, salt, low-temperature, and five herbicides containing mesotrione, florasulam, nicosulfuron, fluroxypyr, and sethoxydim. In summary, our work was the first to include genomic analysis of the SiCYP450 family in foxtail millet. These results provide valuable information for studying the development and stress physiology of Gramineous crops and a prospect for genetic improvement.

## Data Availability

Data are included in the article and Supplementary Materials.

## Supplementary Materials

The supporting information can be downloaded at: https://www.mdpi.com/article/10.3390/ijms241311053/s1.
