# Supplementary figures and images for: Genome-Wide Identification and Expression Profiling of Cytochrome P450 Monooxygenase Superfamily in Foxtail Millet
Source: Int J Mol Sci. 2023 Jul 4;24(13):11053. doi: 10.3390/ijms241311053 (PMC10341998; doi:10.3390/ijms241311053)

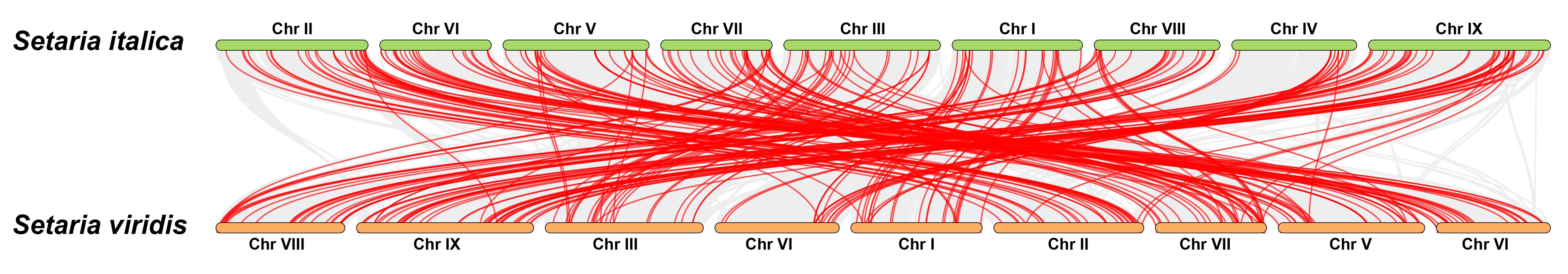

Supplement: Supplementary file 1 [file ijms-24-11053-s001.zip › Supplementary Files/Figure S2. Synteny analyses of CYP450 genes between Setaria italica and Setaria viridis.jpg]

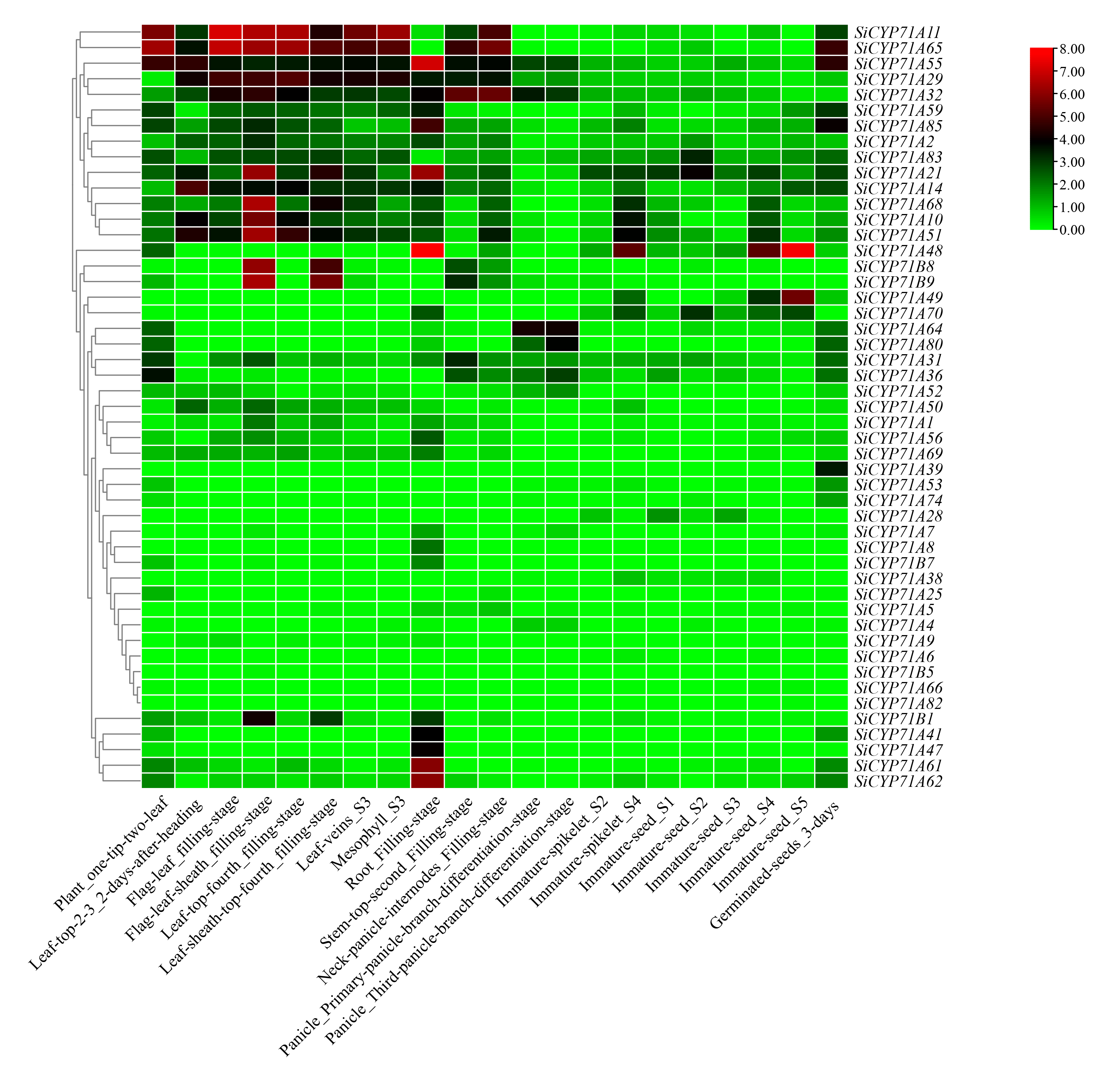

Supplement: Supplementary file 1 [file ijms-24-11053-s001.zip › Supplementary Files/Figure S3. Relative expression patterns (FPKM value) of some CYP7X group genes involved in 21 tissues and different developmental stages of foxtail millet.png]

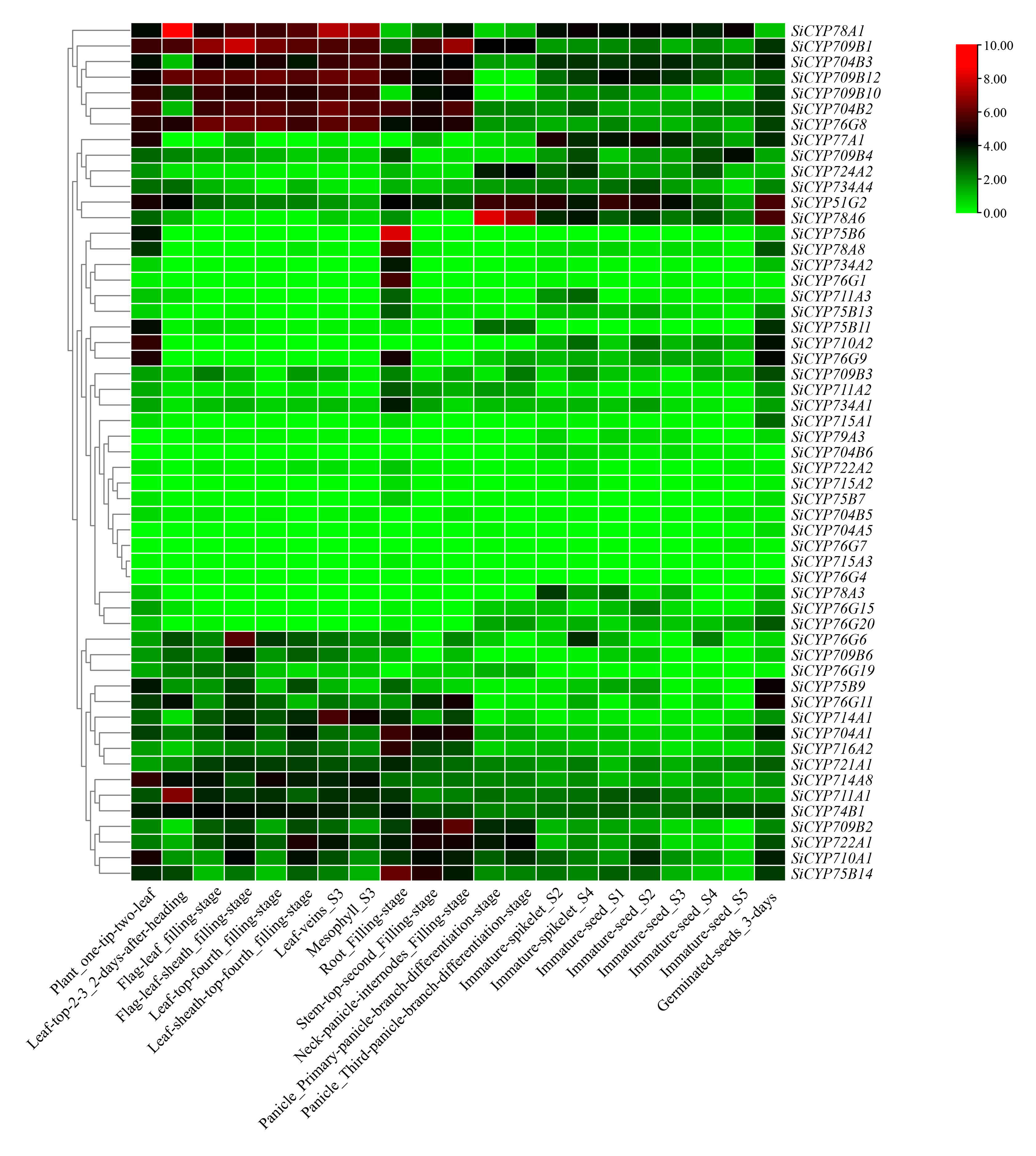

Supplement: Supplementary file 1 [file ijms-24-11053-s001.zip › Supplementary Files/Figure S4. Relative expression patterns (FPKM value) of some CYP7X group genes involved in 21 tissues and different developmental stages of foxtail millet.png]

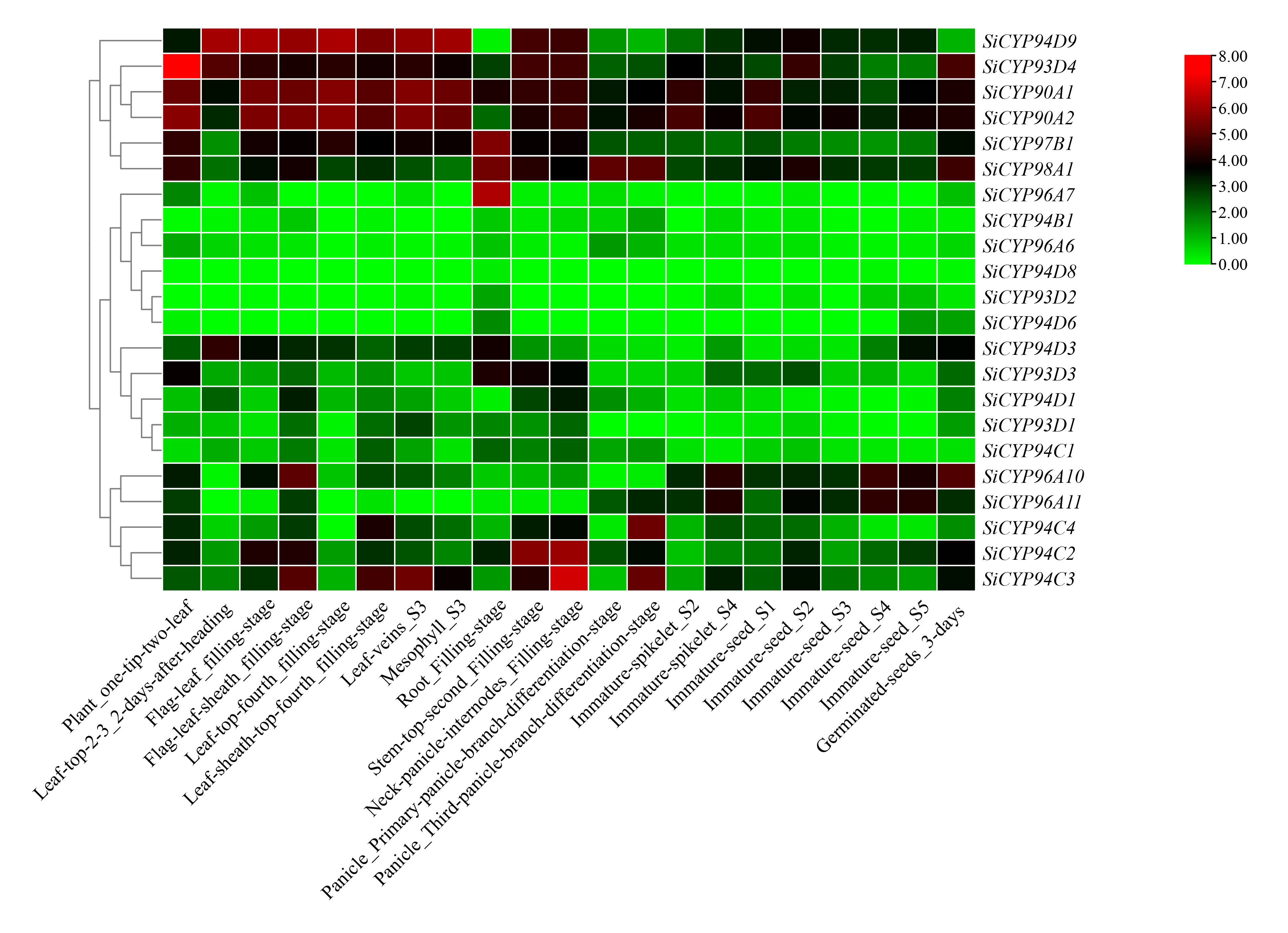

Supplement: Supplementary file 1 [file ijms-24-11053-s001.zip › Supplementary Files/Figure S5. Relative expression patterns (FPKM value) of CYP9X group genes involved in 21 tissues and dif-ferent developmental stages of foxtail millet.png]

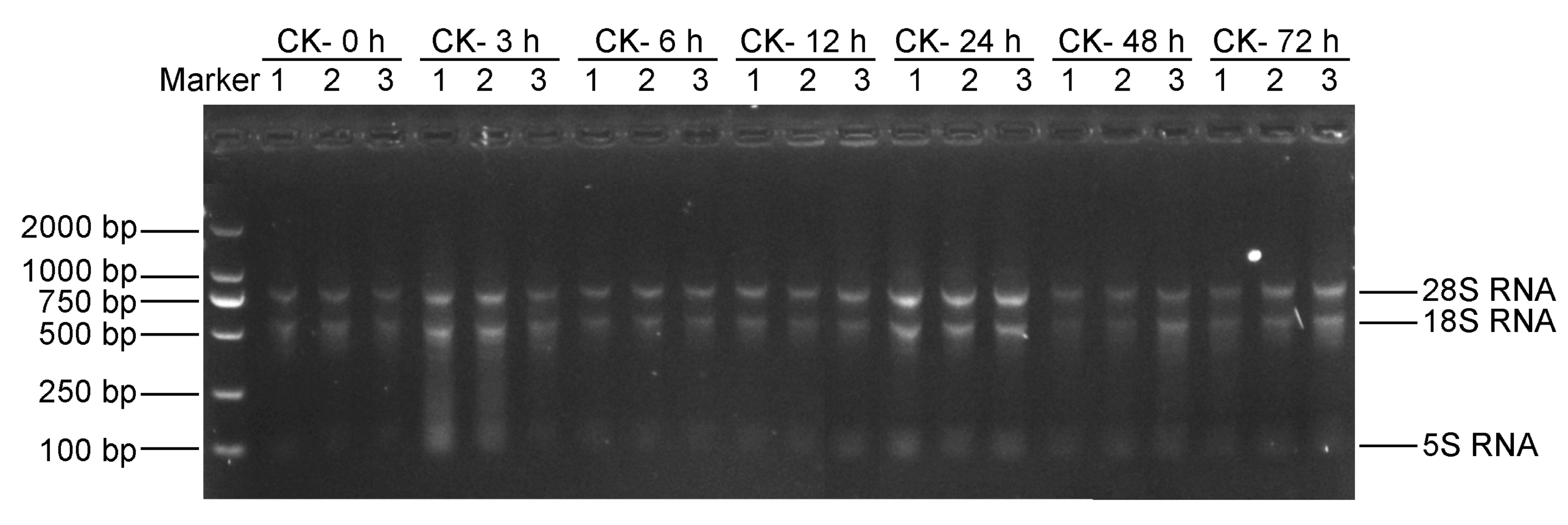

Supplement: Supplementary file 1 [file ijms-24-11053-s001.zip › Supplementary Files/Figure S6. The RNA bands in agarose gels of the non-treated foxtail millet plants.jpg]
